# The Human Need for Equilibrium: Qualitative Study on the Ingenuity, Technical Competency, and Changing Strategies of People With Dementia Seeking Health Information

**DOI:** 10.2196/35072

**Published:** 2022-08-11

**Authors:** Emma Dixon, Jesse Anderson, Diana C Blackwelder, Mary L Radnofsky, Amanda Lazar

**Affiliations:** 1 College of Information Studies University of Maryland College Park, MD United States

**Keywords:** dementia, health information behavior, action research, equilibrium, postdiagnostic experience, mobile phone

## Abstract

**Background:**

Prior research on health information behaviors of people with dementia has primarily focused on examining the types of information exchanged by people with dementia using various web-based platforms. A previous study investigated the information behaviors of people with dementia within a month of their diagnosis. There is an empirical gap in the literature regarding the evolution of health information needs and behaviors of people with dementia as their condition progresses.

**Objective:**

Our work primarily investigated the information behaviors of people with dementia who have been living with the condition for several (4 to 26) years. We also aimed to identify their motivations for changing their information behaviors over time. Our primary research questions were as follows: how do people with dementia get informed about their condition, and why do people with dementia seek information about their condition?

**Methods:**

We adopted an action research approach by including 2 people with dementia as members of our research team. Collaboratively, we conducted 16 remote 1-hour contextual inquiry sessions with people living with mild to moderate dementia. During the study sessions, the first 40 minutes included semistructured interviews with participants concerning their information behaviors, followed by a 20-minute demonstration of their information-seeking strategies. Data from these interviews were analyzed using a constructivist grounded theory approach.

**Results:**

Participants described their information needs in terms of managing the disrupted physiological, emotional, and social aspects of their lives following a diagnosis of dementia. They used various information behaviors, including active search, ongoing search, monitoring, proxy search, information avoidance, and selective exposure. These information behaviors were not stagnant; however, they were adapted to accommodate the changing circumstances of their dementia and their lives as they worked to re-establish equilibrium to continue to engage in life while living with a degenerative neurological condition.

**Conclusions:**

Our research revealed the motivations, changing abilities, and chosen strategies of people with dementia in their search for information as their condition evolves. This knowledge can be used to develop and improve person-centered information and support services for people with dementia so that they can more easily re-establish equilibrium and continue to engage in life.

## Introduction

### Background

Information behavior refers to the ways in which people interact (and do not interact) with information [1]. Understanding health information behavior is important as decisions based on health information sources can influence a person’s health trajectory, quality of life, and health outcomes [1,2]. Populations with unique health information needs may exhibit different health information behaviors. Therefore, researchers have studied the information behaviors of populations such as people with diabetes [3,4], breast cancer [5], and prostate cancer [6]. We studied the health information needs and behaviors of people with dementia as, similar to those with other health conditions, they want to be fully informed about their diagnosis [7-10]; however, the cognitive nature of their medical condition makes this inherently difficult. Therefore, we must identify the information that they seek, as well as find the most appropriate ways to provide it, to meet their needs.

To date, nearly all dementia-related information behavior research has analyzed data created by people with dementia using social media (eg, Twitter [11,12] and Facebook [13,14]), web-based dementia forums [15-17], and a web-based dementia advocacy platform to share their experiences [18]. These studies revealed the importance of web-based communities for information exchange among people with dementia, although they did not explore the broader information behaviors of people with dementia.

Most research on health information research on dementia has focused on informal caregivers’ information behaviors. A recent scoping literature review of 20 studies found 4 studies on the information needs and information-seeking behaviors of people with dementia, with only 2 studies distinguishing the behaviors of people with dementia from those of caregivers [19]. Studies that delineate the unique information needs and information behaviors of people with dementia found that caregivers were more interested in searching for topics specific to dementia, whereas people with dementia predominantly searched for support groups and were generally disinterested in seeking information regarding dementia [20, 21].

Harland et al [10] found that perceptions of dementia were major factors affecting information-seeking behaviors immediately after diagnosis. For example, people with dementia avoided health information if they felt unable to influence their situation, whereas others valued and sought this information to “confirm their suspicions and provide explanations” [10]. Harland et al [10] called for further work to understand “how information needs and behaviors change over time and with the progression of the disease”—a call to which this paper responds.

### Objective

The primary aim of this study was to discover the nature, content, and evolution of information behaviors of people living several years after a dementia diagnosis. Our secondary aim was to identify the motivations for changing information behaviors over time. Therefore, we sought to address the following research questions:

1. How do people with dementia stay informed about their condition?
2. Why do people with dementia seek information about their condition?

To answer these research questions, we adopted an action research approach [22], which included contextual inquiry sessions with 16 people with dementia to understand the factors that motivated them to initially seek information. We then examined their evolving information behaviors and investigated their motivations for adapting their information behaviors. In addition, our findings led to revelations beyond the scope of our original interview questions, which unveiled information behaviors in the realms of personal well-being and equilibrium. Equilibrium, defined as a state of balance between a person’s mental framework (cognitive and emotional) and the environment [23], is a concept that has been applied in domains ranging from education to health sciences. Specifically, researchers have noted ways in which people strive for equilibrium when coping with a new medical diagnosis [24,25], which triggers disequilibrium, a state of disorder and imbalance in the mind [23]. Past research has noted how individuals with conditions such as cancer [24] and diabetes [25] actively search for information as a way of restoring equilibrium. In our study, several kinds of information behavior, including actively searching for information, played a key role in restoring equilibrium in participants with dementia. We compare our findings to prior research and discuss the implications of our findings for medical professionals who work with people with dementia, as well as health information resource providers such as web-based content developers interested in creating more accessible content.

## Methods

### Study Overview

Our research team comprised university researchers and 2 dementia advisers (people living with dementia who are knowledgeable and active in dementia advocacy). We selected an action research approach [22] that involved members of the target culture throughout the research process [22]. The 2 dementia advisers on our team served as empowered researchers and decision-makers. The participation of dementia advisers afforded the research team greater access to otherwise hidden truths in the population of people with dementia. The research team collaboratively determined the topic of the study, identified target questions, designed the study protocol, collected and analyzed the data, and wrote the final report.

A contextual inquiry approach [26] allowed us to collect data through participants’ verbal explanations, as well as their demonstration of information-seeking behaviors. This strategy gives participants the chance to explain and attempt to perform their intentions, thereby not limiting data collection to recall or verbal descriptions of information behaviors [26,27]. This is particularly useful when conducting research with people with cognitive disabilities. We chose a qualitative approach rather than using a priori theories and methodologies to describe complex, dynamic participant experiences.

### Recruitment

We recruited people with dementia through convenience sampling from our networks, which included members of peer support groups, large dementia advocacy organizations, and snowball sampling. To qualify for the study, participants had to self-report a clinical diagnosis of mild to moderate dementia and seek dementia information since being diagnosed. The research team first sent 30 potential participants an initial recruitment email with details outlining the study and the criteria for participation in the study. Of the 30 participants, 16 (53%) responded that they met the participation criteria and were interested and available to participate. Before scheduling each study session, the authors verified the eligibility of the participants by verbally inquiring about the participation criteria. Interview and observation sessions were scheduled and completed between July and September 2020.

### Data Collection

The 16 contextual inquiries comprised 2 parts: an interview (about 40 minutes), followed by a 20-minute observation session. Sessions were conducted on Zoom videoconferencing (Zoom Video Communications Inc) because of pandemic-related research restrictions. The first 4 contextual inquiry sessions were conducted by a team of 2 (ED and JA) academic researchers. The remaining 12 contextual inquiry sessions were conducted by blended teams (1 academic researcher [ED teamed with DCB] and 1 dementia adviser [JA teamed with MLR]), with the dementia adviser leading each session.

### Ethics Approval

We assumed consent capacity, which aligns with best practices when working with people having mild to moderate dementia [28] and is required by law in many countries [29]. As a precaution, we were attentive to a possible lack of capacity and were prepared to use the University of California Davis protocol [30] to determine whether the person was able to do the following: (1) make a choice to participate, (2) show an understanding of what the study entailed, (3) describe their rationale for participating in the study, and (4) show an appreciation of the potential risks and benefits of the study. This protocol was not used for any participants, as there was no indication that any participant might not have the capacity to consent.

In each contextual inquiry session, participants first provided verbal consent and then completed a short demographics form. In semistructured interviews, we asked questions such as “How do you typically obtain dementia-related information?” and “How would you improve your process of obtaining dementia information?” We subsequently followed up with probing questions to pursue topics raised by the informants (eg, conversations with their physicians). During each observation, the screen-sharing technology allowed participants to demonstrate their information-seeking strategies.

On the basis of our related work involving people with dementia, we created 8 scenarios as prompts for information seeking (see Multimedia Appendix 1 for scenarios), and then invited participants to select and conduct a search while describing their intentions. During this phase, we probed participants to reveal their thought processes using questions such as “Why did you choose this site?” Following each session, participants received US $20 Amazon gift cards as compensation.

Raw data comprised session transcriptions, screen videos of web-based searches, field notes, methodological memos, and Zoom video and audio recordings, which resulted in approximately 16 hours of recorded data. This was transcribed using Otter.ai (AISense, Inc) and reviewed and verified by an academic researcher. All procedures were approved by the University of Maryland institutional review board (approval number 1316631-40).

### Data Analysis

We used a constructivist grounded theory approach to analyze the interview data [31] as it required taking into consideration and accounting for our own perspectives as researchers. This was critical, given that our research team included both dementia advisers and academic researchers.

Each of us open coded the transcriptions, creating early codes such as “offline information gathering strategies” and “vetting people/organizations.” We collaboratively discussed our codes and determined emergent themes, such as “information sources and systems,” “information gathering strategies,” and “motivations for seeking information.” Individual codes were merged into collaborative themes, resulting in a code book. The team then collectively edited, clarified, and refined the code book. The resulting code book was applied during the refining process of focused coding using the most significant initial codes to sift through large amounts of data to categorize the data incisively and completely [31].

Each transcript was focus coded twice—once by a dementia adviser and then by an academic researcher—to ensure that we correctly understood what each participant’s intended meaning. Through this process, our analysis led us to understand how individuals use different types of information behaviors, which has been previously defined in the literature (although, to the best of our knowledge, not in the context of dementia).

We followed an iterative process of engaging with the data, comparing codes, performing pattern recognition, and memoing for several months with weekly team meetings to build connections between codes and emergent theories that would explain the nature of participants’ health information–seeking behaviors. This process led to a dementia adviser connecting the concept of “finding equilibrium,” based on the theory of “optimizing re-equilibration” [23], to the motivation that participants described for their information-seeking strategies and their subsequent emergent changes in information behaviors, which we describe further in the results.

When studying the lived experiences of individuals or groups of people, we may (consciously and unconsciously) superimpose our own perspectives, cultures, and experiences onto the analyses [31]. Therefore, we explicitly reveal our beliefs and status as academics or people living with dementia, so that the reader may interpret our findings with a full understanding of our roles [31]. Although based at a research university, the academic researchers have also been informed by dementia activists who advocate for people with dementia and are known to practice self-determination well into the progression of the condition [32-34]. In addition, technology research has informed our practice of supporting people with dementia [35] and other disabling conditions to take an active role in their own health and well-being [36-39]. These perspectives have influenced the ways in which we conduct research on how people with dementia use information.

### Participants

A total of 16 participants aged 57 to79 years self-reported at least one type of mild to moderate dementia with a range of 4 to 26 years since their diagnosis before the study (Table 1). Of the 16 participants, 15 (94%) identified ethnically as White and 1 (6%) as Asian. All participants reported employment statuses of retired, retired on disability, or volunteering. Nearly all claimed being “somewhat confident” using technology (Thomas was “not confident,” whereas Lila, Lucy, and Carter were “very confident”). All participants were dementia advocates and members of online peer support dementia groups.

**Table 1:** Participant demographics.

Pseudonym	Age (years)	Sex	Country of residence	Type of dementia	Years since diagnosis	Education	Technical confidence
Arnold	68	Male	Canada	Vascular dementia	≥10	Some college; no degree	Somewhat confident
Dawson	73	Male	United States	Early onset Alzheimer disease	2 to 5	Some college; no degree	Somewhat confident
Lucy	67	Female	United Kingdom	Early onset Alzheimer disease	5 to 10	Bachelor’s degree	Very confident
Carter	61	Male	United Kingdom	Vascular dementia	5 to 10	Some college; no degree	Very confident
Michael	61	Male	United States	Functional neurological disorder	5 to 10	Some college; no degree	Somewhat confident
Sadie	79	Female	United States	Alzheimer disease	≥20	High school diploma	Somewhat confident
Lila	Range 60-70	Female	Canada	Early onset Alzheimer disease with a Lewy body component	5 to 10	Multiple bachelor’s degrees	Very confident
Carly	62	Female	United States	Frontotemporal dementia	≥10	Master’s degree	Somewhat confident
Gale	71	Female	Australia	Frontotemporal dementia	5 to 10	Master’s degree	Somewhat confident
Eva	57	Female	United States	Lewy body dementia with behavioral disturbances	5 to 10	Bachelor’s degree	Somewhat confident
Levy	61	Male	No answer	Lewy body or Parkinson disease	2 to 5	Some college; no degree	Only a little confident
Velma	61	Female	Canada	Vascular dementia	5 to 10	Bachelor’s degree	Somewhat confident
Thomas	68	Male	United States	Variant of slow-moving Alzheimer disease	5 to 10	High school diploma	Not at all confident
Kevin	79	Male	United States	Frontotemporal dementia	≥10	Master’s degree	Somewhat confident
Toby	61	Male	United States	Early onset Alzheimer disease and Lewy body	5 to 10	Master’s degree	Somewhat confident
Donna	62	Female	Australia	Semantic variant of primary progressive aphasia	≥10	Master’s degree	Somewhat confident

## Results

### Overview

We discovered the information needs of people with dementia concerning the physiological, emotional, and social aspects of their life. These included the factors that motivated them to seek information on their condition to re-establish equilibrium, both initially after diagnosis and in evolving ways over the years (or decades, in some cases) through active searching, monitoring and ongoing searching, proxy information search, information avoidance, and selective exposure.

### The Goal of Health Information Seeking: Re-establishing Equilibrium After Diagnosis

#### Overview

Disequilibrium is defined as a state of disorder and imbalance in the mind such that individuals cannot assimilate new information into their schema because of its contradictions or inconsistencies with their prior knowledge or experience [23]. In the instances described by the participants, disequilibrium arose with a medical diagnosis of dementia. After hearing that they had an incurable brain condition, participants described feeling sometimes relieved at receiving an actual diagnosis but also still feeling uninformed, confused, worried, sad, upset, or alone in terms of the physiological, emotional, and social aspects of life. As they strived to resolve this disequilibrium, they often did so by seeking relevant health information; however, just as often, they did not find what they needed.

#### Information Needs Concerning Physiological Aspects of Life

Upon diagnosis, participants usually expected physicians to satisfy their information needs regarding their physiological health. However, as nearly all respondents explained, most of their physicians (and even local dementia organizations) failed to provide adequate information regarding the origin, progression, treatment, and management of dementia, both initially and during the evolution of their condition. Arnold explained, “I wasn’t given any referrals, any information, who to talk to, where to go.” Donna, too, recounted as follows:

I was told, “The type of dementia you’ve got, there’s nothing I can do, and no medication available.”...And then, “See you in six months.”

This lack of information at diagnosis led to a state of disequilibrium for participants, as they were unsure of the physiological factors related to what to expect out of life with their new dementia diagnosis.

Even after realizing that their questions might yield unpleasant answers (or none at all), several respondents persevered in researching the nature of their condition. Gale explains this as follows:

When I was first told the diagnosis, I didn’t really know anything...I really wanted to study [frontotemporal dementia], find out what it was, and confirm [to] myself that I thought the symptoms matched.

Participants asked the following:

...medical, mental kinds of stuff...a cure

Kevin

Is this being caused by the [reduced] blood flow?

Velma

Have I taken the proper steps?

Levy

By educating themselves about the physiological aspects of dementia, some participants hoped to learn how to mitigate the effects of dementia on their lives as a way of re-establishing equilibrium. Lila explained the following:

I’ve come to realize that there are different forms [of dementia], and each form comes with a different set of problems. And if I know the problem, maybe I can avert the long term effects.

#### Information Needs Concerning the Emotional Aspects of Life

The emotional toll of the information provided at diagnosis often left the participants hopeless or in an extremely low emotional state. When Velma received a diagnosis of vascular dementia, her physicians told her, “There was nothing that they could do, nothing that they really had for me, and that I needed to get my affairs in order to try to enjoy my window of time.” This led her to an emotional “state of numbness and shock for probably a good six months or so...I couldn’t even figure out what questions I needed to actually ask, because all that kept going through my head was three to eight years,” which is the reported average life expectancy for individuals with vascular dementia. Similarly, Thomas described his diagnosis as “excruciatingly painful to hear,” leading him to “go home, and then you put a blanket over your head, start having some very dark thoughts about...what’s the point of carrying on, if this is all I’ve got?” He added the following:

A lot of health practitioners...don’t practice good emotional care. And emotional care is every bit, if not more important, particularly when you're being told you have a fatal disease.

Participants had previously expected a different trajectory for their lives. Therefore, the diagnosis of dementia was emotionally destabilizing.

Participants searched for encouraging health information to balance the negative news they had received with any positive news—sometimes just to find hope to re-establish equilibrium. However, Thomas realized he was not the best person to do the research:

We need...the wisdom of what to do with this new diagnosis as [physicians] give the diagnosis, not send people home and let them go looking for it.

Disequilibrium upon diagnosis was compounded by failed expectations that physicians would not only fully address his physical and cognitive condition but also his emotional state of being, as it related to the changes he was undergoing.

Other participants experienced not only receiving pessimistic information from their physicians but also a dehumanizing delivery of their diagnosis. Lila’s neurologist “never looked at [her] once.” Instead, the physician announced to her husband, “‘Your wife has early onset Alzheimer's. You can bring her back when she can’t dress herself.’” Lila was furious:

I had to keep my mouth shut. I was biting my tongue so hard, I thought the blood was gonna start pouring out of my mouth...

She later called her primary physician and was referred to another specialist. These kinds of interactions with physicians could last for years, which is explained by Donna as follows:

I had a very good rapport with [the doctor], but after the dementia diagnosis, he wouldn’t see me on my own. After about three years, I took a piece of paper with big type, big black font on it: “Talk to Me.”...He said, “What do you mean?” I said, “Well, before dementia, you used to talk to me about my health. Now you'll only talk to my husband; it’s like I don’t even exist in the room.”

Such dehumanizing interactions with physicians at diagnosis left at least one participant yearning for moral support and information “that touch the person’s heart” (Thomas).

#### Information Needs Concerning Social Aspects of Life

Participants said that their social lives changed from the moment they shared their diagnoses, reporting an apparent lack of empathy from friends or family members who had no prior experience with dementia. Thomas explained the following:

When you finally work up the nerve to tell people what you’re going through, the first thing out of their mouth, 95% of the time, isn’t empathetic. It’s like, “You don’t look like you have dementia! At least you don’t have cancer”...[A spouse] loves you dearly, but doesn’t get the fear that courses through your veins

Lacking a sense of belonging to a given community also contributed to the participants’ state of disequilibrium, driving them to seek information that would help with the social aspects of their lives.

Participants described the need for social connections with other people with dementia and sought further information on their similar lived experiences:

I knew that I couldn’t be the only one...So I was searching—where are these people? I have to find them. I know they’re out there

Velma

Connecting to other people with dementia was key to understanding how to live well; knowledge from their lived experiences could not be gleaned from other relationships. Lila described the following:

We need to learn from each other. We need to hear how other people do things and what they’ve gone through.

Given that many participants could not locate others with dementia nearby, they “had to go online” (Sadie) to accommodate information needs concerning social aspects of life to re-establish equilibrium.

### Evolving Information Behaviors of People With Dementia

#### Overview

Although participants expressed the need for more health information immediately following the diagnosis of dementia, they also described changes in information needs as their medical condition progressed, as new medical discoveries occurred, and as access to different types of information became known. Their initial needs and formerly successful strategies to seek information no longer provided them with equilibrium; therefore, they adapted. At the time of the study, 31% (5/16) of participants had been living with dementia for >10 years, and one participant for even 20 years, and we learned about changes in their information needs and behaviors, along with those of the more recently diagnosed people.

#### Active Searching

##### Overview

The first information behavior most participants described following diagnosis reflected the Wilson active search technique, meaning they were “intentionally choosing to browse or search for information” [40]. Participants indicated that the goal of their active searching immediately after diagnosis aided them in recovering from initial postdiagnostic reactions such as depression or denial. Active searching also took place months and years later from digital resources and other people with dementia in online peer support groups.

##### Digital Resources

As many participants felt they were provided insufficient information at diagnosis, they described actively “searching out every little thing” (Levy) to satisfy their information needs. Carly described herself as “a questioning person...a thinking person,” where she tries to clarify the things she’s read by “immediately look[ing] [it] up.”

Given the multiple types of dementia and their myriad of symptoms, participants had varied physiological information needs, which led them to actively search for web-based resources. Velma, who described herself as “always learning—everyday I’m learning,” read “a lot of new reports that come out in JAMA...I’m learning how important things like nutrition, exercise, socialization and connections with people are, in staying well.” When experiencing potential symptoms of dementia, Lila searched for “‘dementia and left side brain’ So, you know, because of the regions in the brain, depending on which side you have dementia attacks and the areas it attacks, it will also tell you what symptoms could be happening to you.” Other participants wanted to understand whether symptoms, such as loss of smell and taste (Velma), were related to dementia or to another condition that they might need to treat.

Participants routinely conducted active searches on the internet as they knew that research is “evolving all the time” (Dawson). Even after 12 years of living with dementia, Donna looks up new information “all the time, because everything’s changing so much...even stuff about diagnosing categories.” Similarly, Dawson acknowledged that “what was in Google out there 10 years ago, 8 years ago, 5 years ago, 10 minutes ago, is always changing...”

For many participants, the sources of information they used were dependent on the type of information they were trying to find. Carly described: “I have many systems—dependent upon the question...” For basic information, Carly would use the Google search engine. When this was “not enough,” she would turn to peers with dementia. These peer interactions during the process of actively seeking web-based information were typically synchronous and presented in a mutually beneficial manner, as discussed in the following section.

##### Counterparts in Peer Support Groups

Without exception, all participants said that they actively sought information about dementia from others with similar diagnoses in online support groups, citing this as one of the most important strategies for gathering useful information.

Several participants described actively seeking physiological information from other people with dementia who had been living with the disease longer, and seemingly better, through web-based synchronous communication using videoconferencing platforms such as Zoom. For example, Eva and Michael both referred to a web-based social group called “Dementia Mentors” [41], which provides a safe, nonjudgmental, stress-free environment several times a week. Through this program, people recently diagnosed with dementia can communicate with people who have lived with the condition for many years via web-based interactions.

The participants also mentioned organizations that offered online peer support groups, such as the Alzheimer’s Association, Association for Frontotemporal Dementia, Lewy Body Association, Alzheimer’s Society, Dementia Alliance International, and Dementia Australia. After joining a peer support group, Velma “started to have a better understanding of how much there was that we could do to help ourselves.” For Toby, the group provided an opportunity to observe other group members, and “almost [see] a case study in what’s going to happen to me vicariously,” which satisfied his need for information to understand the physiological changes he might experience in the future. Similarly, Dawson explained the following:

You can see they’ve experienced it and you have faith and trust in that individual...if they’ve had a good experience with a particular medication or some activity that they’ve done, then it may be something I might want to consider...we share that information.

Such online peer support groups helped participants identify, and often actually provided, the information necessary to re-establish equilibrium in their lives.

Online peer-to-peer support groups provide a place where participants “share your problems with people who get it...all the different nuances and fears,” similar to “a foxhole connection you get when you’re in war” (Thomas). Information sharing created a bond that “wasn’t the social thing; it was much deeper than that...this connection is heartfelt, emotional, mental and spiritual” (Thomas). This connection helped Thomas to “overcome my own severe negative feelings about people with dementia, and recognize that there’s still life worth living, because I was just like everyone else.” Arnold expressed how “what we say stays in the group, and we’re not pre-judged.” Knowing that everyone is similarly vulnerable and that confidentiality is preserved seemed to make peer groups feel safe, even allowing for personal growth. These web-based communities seem to be highly valued resources for re-establishing equilibrium by meeting the information needs concerning the social and emotional aspects of life.

#### Monitoring and Ongoing Searching

##### Overview

Participants explained how an active search helped them build “a base of knowledge” (Toby) on dementia and re-establish equilibrium by meeting their physiological, social, and emotional information needs after diagnosis. With these needs met, and equilibrium restored, some shifted to searching less often, such as Gale:

In all honesty I tend not to look for anything specific nowadays, because I kind of feel that I’ve got all the basic information.

These participants adopted the information behavior of ongoing search, “where active searching has already established the basic framework of knowledge, but where occasional continuing search is carried out to update or expand one’s framework” [40]. To facilitate an ongoing search, participants set up what Ellis defines as monitoring strategies, which include any actions that enable someone to stay updated with new developments in their field [42]. For example, Gale explained that when “somebody has prompted me with a subscription or said something, I’d say, ‘Oh yeah; I’ll look that up and see.’” When new information was inconsistent with the existing knowledge base of the participants, they would slip into disequilibrium, prompting the need to conduct an ongoing search for further information to re-establish equilibrium.

Monitoring strategies included attending peer support groups, curating social media accounts, and receiving push notifications such as subscribing to newsletters, as discussed in the following sections.

##### Peer Support Groups

Monitoring and ongoing searches in peer support groups of people with dementia were important sources of health information for the participants when “someone else in the group may bring one [research article] forward and go, ‘Hey, did you see this one?’” (Velma). Thomas believed that current clinical research “would come up in group, ‘cause we have people in our group that are proactive about those kinds of things,” alluding to peers who routinely conducted active searches. Arnold, who is in several peer support groups, explained, “I’ve been caught off-guard with some information...These groups are a tremendous source of information and help.”

Lucy occasionally conducted ongoing searches:

I don’t actually go online unless...a discussion has come up and I want to know more about it...I will go on Google Search and find out...a little bit more about what they’re talking about.

She monitors information in the “nine different groups that I’m involved in” with the goal of “shar[ing] all the information I’ve collected from others...[because] it’s important, isn’t it? Because they don’t know, and that’s something I’m still capable of...getting all this information and letting them know.”

##### Social Media

Participants also used social media platforms such as Twitter to monitor rather than actively search for relevant health information. Arnold explained that “About 95% of the people that I follow are dementia-related,” such as researchers and advocacy organizations. He elaborated that he is “not using Twitter to get information; it’s more seeing what people in the dementia world are up to,” reflecting a monitoring strategy. He “look[s] at everything that’s posted. And then I’ll click the heart, which means I like it. And sometimes I will make a retweet with a comment. But that’s basically it.”

Donna checks, “if I see something on Twitter, and there’s a lot of chatter about it, I think, ‘Oh, I better read that one,’” reflecting an ongoing search strategy. Carter curated a network on Twitter, although for the “vast majority” of people he followed, he had also “met personally...[and] can vouch for their credibility.”

##### Push Notifications and Subscriptions

Many participants set push notifications from subscription providers to monitor the latest dementia information from organizations such as the Dementia Engagement and Empowering Program (Carter), Alzheimer’s Disease International (Donna), Lewy Body Dementia Association (Levy), international health organizations (World Health Organization and United Nations [Donna]), medical journals (The Journal of the American Medical Association [Velma] and Neuroscience [Gale]), and medical organizations (the Mayo Clinic [Donna and Velma]). Velma described this strategy as, “just wait for them [newsletters] to show up,” as she did not “know how to find a list of what I’m subscribing to or not,” but did regularly read her email.

The participants remarked that these monitoring strategies resulted in a large volume of new information. Gale “subscribed to any new research...I probably get three or four emails a day that have information about what’s going on with dementia.” Donna subscribed to “loads” of blogs that “come into my inbox on a Monday morning...I very rarely go to them in my inbox” as “I just get too many emails, about 600 emails a day across my different emails.” However, this offer of information did not usually create disequilibrium. Instead, as Donna explained, with certain research organizations, such as the Mayo Clinic, “I get their newsletter, but I rarely read it unless it really jumps out as something that might be super relevant.” Therefore, these monitoring strategies only led to ongoing searches when participants were confronted with new information that was not already part of their existing knowledge base or conflicted with their own experiences.

#### Proxy Information Search

Proxy information search is when “one individual tries to find information on another person’s behalf” [43]. Owing to the progression of dementia and changes in abilities, some participants described having to transition from active or ongoing search to proxy information search to continue having access to the kind of dementia-related information they needed. For example, Toby described decreases in “the speed at which I can assimilate information...and the extent to which I can recall information.” Thomas finds actively searching for information difficult as “I don’t retain stuff really good anymore.” Given that so much information seeking related to dementia took place on the web, active and ongoing search became inaccessible to Carter because of his difficulty with “working on the PC” where “things that I used to be able to do, not that long ago, that I can’t do anymore.” As a result, the physiological, social, and emotional aspects of life were inadequately addressed, often leaving participants in a state of disequilibrium. To re-establish equilibrium, some participants found a proxy to help them search for dementia information, thus accommodating their progressive cognitive disabilities.

To reiterate, the participants’ shift to using the strategy of proxy information search did not appear to be based on a waning interest in dementia information or a lack of questions. Toby explained the following:

I generally have the ability to ask the questions and to know the relevant questions and pose them. But I have lost the ability to do some of the deep research that I normally would have done.

In such instances, several participants referred to their spouses as assuming the role of proxy information seeker by “look[ing] stuff up for me” (Thomas) and being “a conduit for information flow” (Toby). Others relied on mentors (Arnold) and family members (Levy). Thomas also relied on people from his support groups.

The participants expressed much gratitude for the proxy searchers. However, some participants were deterred by the need to transition to greater dependency on information. Levy explained his reluctance to use a proxy but also recognized his own limitations:

I don’t really go out looking. I used to...when I first got diagnosed...

However, giving up the power to search for information was a “struggle...because you feel you’re going to lose everything. And it’s like, now I gotta give this up [searching on his own]. It’s killing me” (Levy).

People with dementia often have activities slowly taken away because of their own changing cognitive abilities [32] or from well-intentioned carers to relieve them from certain responsibilities. In either case, the transition to a proxy search may be seen as yet another activity being taken from them. Although participants noted their disappointment and dissatisfaction with having to rely on others to assist with information search, they retained an active role in choosing when to engage in a proxy search.

Some participants described serving as proxy researchers for their peers. When Dawson gave dementia-related talks, people asked him questions about specific types of dementia or how to get tested for dementia. Hence, he would do “a little more digging,” then “usually I’ll share with them at a very high level what I have been able to learn, but I will also ask them to check with their own doctor or check with an association to get specific[s].” By contrast, Gale did not simply conduct searches for people but rather “show[ed] them how to do it [look up information] and where to look,” demonstrating for those capable of looking up dementia information but in need of training on where and how to find it.

#### Information Avoidance and Selective Exposure

Some participants expressed how, after a certain point, continuing to search for dementia information led to a state of disequilibrium rather than providing a means to reach equilibrium. Michael said, “I’m burned out. I’m burned out on looking at that stuff—I get it. I get it—it is what it is.” In response, participants chose information avoidance, or “the human tendency to avoid, ignore, and deny information, particularly in the context of health care” [44], to maintain their equilibrium. When Eva experienced degenerative changes because of dementia, she admitted, “I just let it slide by; I don’t care. I don’t want to know.” In these instances, a participant may experience changes inconsistent with prior knowledge or experiences but still choose not to seek information that would help “go beyond his current state and strike out in new directions” [23]. Instead, some participants described avoiding dementia information or only selectively exposing themselves to dementia information, specifically to maintain their equilibrium.

However, information avoidance did not necessarily signify the end of participants’ information seeking but was used more as a mitigation strategy to avoid shifting into disequilibrium because of information overload at a particular moment. Thomas occasionally used proxy search or information avoidance to focus on life and relationships because of the following:

What’s the point? I mean I know there are some people, they’re going to spend the rest of whatever is left of their life worrying about it, stressing over looking stuff up, and I’d rather go play with my dog and go camping and do what I can, while I can, with what time I’ve got left.

Information avoidance allowed participants to deflect negative emotions and, instead, as Thomas explained, engage in “trying to be a person who’s positive and happy and full of joy. And that’s the way I want to live my life; that’s the way I want to be known.”

The perceived high volume of web-based dementia information was also a contributing factor to some participants’ choices to engage in information avoidance. Eva described being overwhelmed and frustrated:

You get too much information sometimes, and you just don’t want to know any more facts and information.

She also expressed dissatisfaction with not being able to find answers to questions, sometimes simply as there were no answers. Thomas was more fatalistic:

What’s the Internet gonna tell me about how you improve your memory?...As far as I know, there’s no real way to improve your memory; you either have it or you don’t.

For some, the response to overwhelmingly negative dementia information was not avoiding it all but rather avoiding only emotionally triggering stories, which is a practice called selective exposure, wherein conscious decisions are made to consume only certain information [45]. Lila minimized information related to the stages of dementia to manage her anxiety:

Once you’re diagnosed...Well, you know, I have dementia, I’m going to decline. I know that...I’m not going to even think about that. You know I have enough to worry about...Why add something else on my plate that could potentially make me more anxious?

However, she continued to actively search for physiological information on her symptoms. In an observation session, Eva declined to explore the scenario to find information on coping with anxiety caused by dementia, saying, “I don’t need to read it. I live it...I live a lot of my life in paranoia and worry and fear.” She then chose a less sensitive scenario and demonstrated her technical skills without having to lose her equilibrium because of the potential emotional toll of the information content.

## Discussion

### Principal Findings

Our findings, based on the semistructured interviews with 16 participants with dementia recruited through convenience sampling, unveiled how the postdiagnostic information needs of people with dementia concerning their physiological, emotional, and social aspects of life motivated participants to actively search for information on their condition. Participants also used the information behaviors of the ongoing search, monitoring, proxy search, information avoidance, and selective exposure as they worked to re-establish equilibrium. These information behaviors were not stagnant but adapted to accommodate the changing circumstances and needs of their lives with dementia to continue to engage in life while living with a degenerative neurological condition.

### Comparison With Prior Work

Past research examining how information behaviors change over time has included people with cancer [46] and diabetes [47]. Eheman et al [46] also specifically investigated how information-seeking strategies change from active to passive searching after people receive cancer treatment. Although cancer causes 10 million deaths a year, and diabetes 1.5 million, dementia now affects >55 million people worldwide, with >10 million new annual cases [48]. This fast-growing population merits investigation into its information-seeking behaviors, especially as there are several forms of dementia. with even those in the medical field being often unfamiliar with the dozens of diseases that can cause this condition. The result is that people diagnosed with dementia have had to learn about their complex symptoms for themselves while trying to live fulfilling lives.

Prior research on the information behaviors of people with dementia found that caregivers were more interested in searching for topics specific to dementia. On the other hand, people with dementia were more interested in finding support groups but appeared altogether disinterested in seeking dementia information [20,21]. Although our research validates the importance of support groups (and provides evidence for their role in filling physiological, social, and emotional information needs), our findings refute this claim of disinterest. Our findings are more aligned with the recent Harland et al [10] study, which highlights the interests of some people with dementia to seek information 4 weeks after diagnosis, whereas others avoided it as they felt they could not affect their situation [10]. Similarly, we found that some individuals, such as Velma, practiced information avoidance for up to 6 months after diagnosis. Some participants’ justification for information avoidance was because they felt emotionally “numb” (Velma) rather than powerless to affect their situation, as reported in the study by Harland et al [10], and even reported the decisions to engage in various information behaviors, including actively seeking dementia information later in their lives. Because of our semistructured interviews with 16 participants with dementia recruited through convenience sampling, we were able to discover the transitions that many participants underwent in their information behavior.

Even participants who described intentionally avoiding dementia information or only exposing themselves to certain topics many years after diagnosis (because of the emotional toll they anticipated) illustrated conscious choices to maintain equilibrium. Such acts of self-preservation and self-care are perhaps a vital stage of effective dementia information seeking because of the pessimistic, overwhelming, and unsatisfactory extant dementia information. This finding provides a contrasting narrative to the typical assumption that people with dementia are incapable of self-regulation [49]. In fact, participants were able to recognize that searching for dementia information at times led them to an unhealthy state of mind; therefore, they adjusted their actions to avoid disequilibrium.

Thus, information avoidance may be worth exploring in future research, as it may inform physicians and support and advocacy organizations in their efforts to deliver more person-centered, positive dementia-related information. When participants chose to seek dementia information, they developed and used their own systems to monitor advances in research, including regular scrolling through curated social media accounts, which is an avenue for the dissemination of self-narratives by people with dementia [11,12]. Our findings also contribute to the field by demonstrating that some people with dementia use social media in the hopes of hearing about new breakthroughs in medical research regarding dementia. Future studies should consider using social media as an additional platform for disseminating information about support services for individuals with dementia.

Our findings depict that the information behaviors of a group of people with dementia differ from those studied in previous research, which found that people with mild cognitive impairment and dementia underuse social media and push notifications to access health information [50]. Although prior research reported apathy toward technology in the search for web-based dementia resources [51,52], individuals in this study demonstrated a wide range of technology savviness and interest, not only in finding web-based dementia resources but also in setting up monitoring strategies through social media and subscriptions.

We also discovered that participants regularly used web-based platforms to seek out peers with dementia on Twitter [11,12], Facebook [13], dementia advocacy websites [18], and web-based forums [15-17], echoing the importance of web-based communities. Notably, we found that people with dementia seek peers who have lived with the diagnosis longer than they had in synchronous support groups such as “Dementia Mentors” [41]. However, participants also leveraged technology to support others as friends and mentors, with one participant (Gale) teaching newly diagnosed people how to search for dementia information instead of merely serving as a proxy. Such mentors fit the concept of “Dementia Trailblazers” outlined by Johnson et al [17] in 2020—people with dementia who were “extremely active” and knowledgeable in web-based dementia forums in providing information support. Thus, our findings provide evidence of the value of creating support services that incorporate peer mentors to teach people newly diagnosed with dementia how to search for information to meet their physiological, social, and emotional information needs.

Our research on the evolving information behaviors of people with dementia has shown that their search strategies change over time, not only because of the degenerative nature of their condition but more importantly because they remain motivated to re-establish a sense of equilibrium in their lives. Past work has described how people living with cancer [24] and diabetes [25] strive for equilibrium while coping with their diagnosis. It is noteworthy that our study has shown that people with cognitive disabilities are motivated by the same human need for equilibrium in the physiological, social, and emotional aspects of life. To the best of our knowledge, our work is the first to theorize how information behaviors, and the transitioning between them, are motivated by the need to re-establish an equilibrium for people with dementia. Although past work has largely focused on the various motivational factors for information seeking or information avoidance [4,46,47], our findings enrich the health information behavior literature by illustrating a single cross-cutting motivational factor for the range of information behaviors—equilibrium.

### Implications

Revelations from this research will serve to inform medical professionals and web-based content developers about the information needs of the 55 million people with dementia worldwide [48], not only to help them regain equilibrium after the initial diagnosis of a serious brain disease but also to continue supporting them throughout the progression of their condition as they undergo changes in physiological, emotional, and social aspects of their lives.

#### Medical Professionals

Participants described their initial diagnosis of dementia as the trigger to disequilibrium, a finding consistent with prior research that revealed the overwhelmingly negative manner used by physicians to inform patients of their condition, which led to multiple calls for physicians to adjust the language they use to communicate a diagnosis [10,53,54]. Therefore, we add our voices to the call for physicians to convey information in a way that is emotionally reassuring [10] and to “instill hope in the context of a dementia diagnosis” [54].

Left in an information desert after diagnosis, participants were forced to go in search of answers about their type of dementia on the web using only their random technological skills, general education, and access to computers to sift through approximately 1.2 million TB of internet content (as of 2021). Although many participants eventually found health information on the web, it was often not exactly what they needed, took an inordinate amount of time, and was easily lost because of the complexity involved in saving digital information. To combat these challenges, we join in the call of previous work for interprofessional education [55] and multidisciplinary care teams [56] to provide more effective postdiagnosis support to people newly diagnosed with dementia [55]. Our findings demonstrate the necessity of providing such information upon diagnosis in multiple formats and languages (eg, printed, web-based, verbal, pictorial, audio, and video) and when the newly diagnosed individual is emotionally more ready for it. Further work is needed to identify the full range of barriers that people with dementia may encounter when searching for health information on the web.

#### Web-Based Content Developers

In pursuit of dementia information on the web, participants described using social media platforms, search engines, dementia advocacy websites, medical organizations, journal websites, and online peer support groups, although they encountered challenges with each. To address some of these challenges, we ask content developers to better support the information needs of people with dementia in the following areas.

As many participants wanted to actively, although selectively, search for dementia information (to limit their exposure to certain dementia-related topics), we would suggest improving signposts to the content of pages, so that end users can more easily avoid their personal informational triggers to disequilibrium.

Furthermore, the nuisance of subscriptions was frequently discussed, despite the participants’ stated desire to stay informed about health information. A potential solution could be to filter more finely any available web-based content, such as excluding paid advertisements, providing only open-access articles, or showing only reports whose accuracy has been academically verified.

Finally, because of executive function challenges (eg, speed of retrieval, recall, and retention), people with dementia sometimes resorted to proxy searches, which often led to negative emotions around the sense of losing agency. Therefore, we refer to web-based content developers to learn and address the cognitive accessibility needs of neurodiverse end users [57]. These include but are not limited to providing clear and understandable text, using simplified layouts to facilitate the locating of needed information, reducing extraneous material such as advertising and flashing notifications to maintain focus, and offering support for different ways of understanding content [57].

### Limitations

In designing a study involving people with dementia, we were cognizant of the need to respect the time commitment and amount of work we should require. Therefore, we limited the scope of data collection to a single, approximately 1-hour session. We realize that a longitudinal study of participants’ information-seeking behaviors would yield much more data; thus, we hope that future ethnographic or autoethnographic studies will follow the progression of people with dementia, as well as the evolution of their information behaviors.

Participants in this study were limited in racial, geographic, linguistic, and age diversity because of the nature of our convenience sampling recruitment strategy and access to volunteers. All but 1 participant identified as White, and all resided in the United States, the United Kingdom, Canada, or Australia and spoke English, which does not represent the global population currently living with dementia. Prior research shows a higher prevalence of dementia in the United States in African American and Latinx communities [58]. Therefore, future work is needed to ensure that our understanding of information behavior is more representative.

Given that 50% (8/16) of our participants were aged ≤65 years, we also acknowledge an overrepresentation of people with younger onset dementia [59], which only accounts for 9% of global diagnoses [48], although this number may be an underrepresentation of actual cases. Recruiting this relatively younger group of participants may have resulted from the hesitation of the older generation to reveal their diagnoses because of stigma [32,60,61] and misinformation about dementia, which leads to an unwillingness to discuss personal experiences with researchers [62]. The overrepresentation may also have been because participants were recruited using convenience sampling from the most visible peer support and advocacy groups that regularly expressed an interest in helping dementia researchers. Finally, most participants in this study used technology confidently, which may not be representative of the general population with mild to moderate dementia, although this is trending upward: 54.14% of people with mild cognitive impairment or dementia report using their smartphones and tablets almost daily [63].

### Conclusions

Using an action research methodology with 2 dementia advisers and academic researchers, we collaboratively identified the motivations and evolution of the information behaviors exhibited by people with dementia after diagnosis. Participants demonstrated their ingenuity and changing abilities to search for information themselves, shifting their information behaviors (eg, from active to monitoring or proxy searches and from ongoing searches to information avoidance and selective exposure), and discussing such changes to maintain and re-establish their sense of equilibrium in life.

Participants also demonstrated and reflected on their abilities, challenges, and frustrations when seeking dementia information on the web. We found that people with dementia (similar to people with cancer, diabetes, and other conditions that trigger disequilibrium) were motivated to adapt their information behaviors not only to meet their evolving cognitive needs but also to address the physiological, emotional, and social aspects of their lives to re-establish equilibrium when faced with inconsistent or conflicting information, even as their condition progressed.

By informing physicians, support organizations, web-based health resource developers, and other factors that influence information behaviors of people with dementia, we aim to see innovation in communication technologies and media platforms to facilitate their use by a neurodiverse population. In face-to-face interactions, we hope that groups will incorporate peer mentors to teach people newly diagnosed with dementia how and where to search for trustworthy health information on the internet, as well as from their medical providers. We also anticipate that our findings will guide readers newly diagnosed with dementia and who are experiencing disequilibrium to try some of the participants’ strategies to search for the web-based health information that they need to re-establish equilibrium in their own lives.

## Appendix

Multimedia Appendix 1 Semistructured interview and observation guide used to understand information search by people living with dementia.
