# Supplementary material for: The Human Need for Equilibrium: Qualitative Study on the Ingenuity, Technical Competency, and Changing Strategies of People With Dementia Seeking Health Information
Source: J Med Internet Res. 2022 Aug 11;24(8):e35072. doi: 10.2196/35072 (PMC9412905; doi:10.2196/35072)
Supplement: Multimedia Appendix 1 [file jmir_v24i8e35072_app1.pdf]

Multimedia Appendix 1: Semi-Structured Interview/Observation Guide to Understand Information Search by Persons Living with Dementia (PLwD)

Introduction

Thank you for participating in our study. The goal of the study is to learn about how you search for information online to help you understand dementia and/or to guide your new life post diagnosis.

The study session is anticipated to take approximately one hour. The study session will be audio/video recorded. Information collected during your participation will be kept anonymous. You have the right to stop participating in the study, for any reason, at any time. Before the study session, you will be asked to complete a consent form and a basic demographic form if you wish to proceed.

The study session will involve three stages:

1. First, we will verbally discuss the strategies you use to finding dementia related information.
2. Second, a list of different scenarios of information needs common to PLwD will be given to you. You will then chose a few scenarios and search to find information online to address the need presented in the scenarios you choose. You can use whatever online resources you would like.
3. Third, while you are searching for the information in stage 2 we will interact with you to allow you to convey your thought process and decision making processes in real time.

Initial Interview Questions:
Opening Questions Defining Dementia Related Information:

- How would you define dementia related information? What types of things do you think of when you hear “dementia related” information?
    - Are they defining it as illness focused or more related to your daily lived experiences? [*e.g. MayoClinic; symptoms and treatment vs. health forums connecting people about shared experiences living with dementia*].
- What types of information were you seeking before diagnosis?
- What information were you given by your physician when originally diagnosed?
- What questions or concerns did you need information on immediately after diagnosis? [*this will help inform how much research was even necessary. For example, even if only minimal info provided if that info calmed the person and left them feeling informed or if it left them feeling helpless both could result in the lack of desire to look for further information*.]
- After you got that information were you interested in anything else?
- Do you still find the need to look up or learn new things about living with dementia or do you just go about life learning from your own experiences?

Questions about their process:

- How do you typically obtain dementia related information? [*e.g. books, peer support groups, online forums, google, Facebook, twitter*]
    - Same question re-worded: What are your preferences for information sources? [*online, in person, medical professional, support groups, Alzheimer's Association or other non profit dementia specific organizations*]
- Do you use any commercially available technologies for information retrieval to manage your condition? [*e.g. Google, online forums, dementia advocacy organizations, etc*.]
- Are there any other ways you obtain needed information that are different from the typical ways you just described?
- Do you subscribe to dementia related newsletters, blogs, podcasts, etc? [*Probe for passive receiving of information vs. actively searching for information*].
- Are you a member of an in person or online peer or other dementia related support group?
    - If so, is this a source of your dementia information retrieval?
    - Have members of your group ever suggested different online informational sources?
    - If so, did you find these sources were helpful?
- Do you typically just browse for information online, meaning you don't go in looking for something specific? Or do you go online with a specific dementia informational need in mind (*e.g. searching for a specific type of medication*)?
    - How does your process look different when you are simply browsing versus when you are searching for specific information?
    - Which do you prefer, browsing or searching directly and why?
    - How have the changes you've experienced with memory or attention span affected your ability to browse or search for information?
- How often do you use online resources to find information to help you understand changes you experience while living with dementia?
    - Are there times when your internet use to search for dementia related information has spiked, such as when you were first diagnosed?
    - Are there costs surrounding this spike in information use (*e.g., the desire to learn as much as possible about their illness at the expense of succumbing to potentially false information in the process or information overload*)?
    - Do you approach searching for information differently during these sudden need for information?
- When you have dementia related questions or concerns do you feel like you're typically able to find answers online?
    - How many additional sources do you generally look at after the first resource you click on? [*probe here for satisficing: opting for choices that are less ideal as long as they are good enough*]
    - What criteria do you use to evaluate whether a resource effectively answers your questions?
- Are you satisfied with your process for finding dementia related information?

- Does your process of finding dementia related information empower you to search for more resources in the future?
- How have you improved your process?
  - Have you come across any common website design for dementia related resources that have made finding information more difficult or easy?
- Think of an ideal information retrieval technology for you. How would you envision it to be?

Scenarios were derived from related work, our past work and conversations on our research team. If possible, scenarios will be reviewed by peer-facilitators with dementia to make sure the wording is sensitive and appropriate and that we are not stereotyping. The scenarios used in the study will look like the example scenarios presented below.

Scenarios will be shared with participants where they will be given the option to choose the scenarios that resonate with them to look up online dementia information.

Some people have expressed how emotionally difficult it can be to view dementia related information. Are you comfortable looking up this information for our observation session?

- Would you want to participate in a focus groups about this topic in the future?
- We want to learn how you search for information online. So we provide a few scenarios for general information concerns of people with dementia. We'd like to observe you looking up information about these concepts. So which scenario would you like to look up online information on?

Scenario 1:
Suppose your doctor diagnosed you with vascular dementia it was such a shock that you couldn't even think to ask any questions about what to expect. It took a few days for you to grieve and now the questions have started to come. Try to find information online about xyz

First, we'd like you to browse the internet to find information on what you should expect now that you have a diagnosis of Vascular Dementia.

Second, try to find information on how to treat vascular dementia and slow the progression of the condition?

Scenario 2:
Suppose navigation has started to become a problem for you. You notice you are more easily confused when trying to go from place to place. Try to find information online to help you better navigate your physical surroundings when you have dementia.

Scenario 3:
Suppose anxiety and worry have become an everyday occurrence for you ever since your diagnosis of dementia. You worry about many things but are wondering if there are any

strategies you could use to overcome this anxiety when it happens. Try to find information online on how to cope with anxiety when you have dementia.

Scenario 4:
Suppose you are consistently forgetting people you know well and see often. Try to find information online to help improve your memory.

Scenario 5:
Suppose you notice that you are no longer the cool tempered individual you used to be. You often find yourself becoming very irritable and angry when you are not able to complete a task that you used to be able to complete easily. Try to find information online if this could be a result of your dementia or perhaps something else and determine what actions to take.

Scenario 6:
Suppose you have started to experience visual spatial issues. You now have trouble doing everyday tasks like mowing the yard correctly, or walking down carpeted stairs with very busy patterns. Try to find information on how to deal with changing vision due to dementia.

Scenario 7:
Suppose after your diagnosis of dementia you are having a hard time telling your family. You aren't sure how to go about the conversation and what you need to be planning for. Try to find information on best practices for disclosing your diagnosis of dementia to loved ones.

Observation Session:
Optional Questions:

- What types of things do you consider or techniques used when deciding whether or not to trust the dementia related information you find online?
- Do you always use this search engine? Why or why not?
- Why did you choose this site?
- Why did you not choose one of these other sites (*probing for specific maybe ads they did not choose*)?
- Why did you decide to do that (*select something, go back, start a new search*)?
- How do you feel using this site?
- What do you like about this site?
- What do you dislike about this site?
- How do you decide if information is trustworthy or not?
- Is this your first time using this resource?
- Do you typically use this website?
- If yes, How did you find out about this? Doctor? Friend?
- How do you feel about the way this information is presented?
- What do you like about this medium?
- What do you dislike about this medium?
- What is your level of trust on the information given by this medium?

- What challenges and/or barriers do you experience when trying to find trustworthy dementia information online?
- Are there any functions this medium does not do that you wish it did?

Things to observe:

- What sort of key-words are they searching? Are they using the words in the prompt or coming up with their own.
- Is this process different when we ask them to just browse dementia related information?
- Are they just looking at titles of websites or organizations?
- How does meta-data influence searching? Does this become more important as we say do a structured search as opposed to browsing, what catches their eye?
- Do they return to websites for information? If so, why?
- Probe to ask if they were returning to confirm the information they found on other websites with this website? If so, what qualities made them see this website as more trustworthy?
- What factors of the online resource make them want to select it (e.g. initial attraction)?
- Upon further inspection of the resource, (follow-up appraisal) what factors affect the trust they feel towards the resource?

Final Questions about the study process:

- Ask participants if they had any comments about the process of the study?
- What did they like or dislike?
- What could we improve the session?
- How could we make it better for future participants?

End of the Study

- Thank the participant for their time
- Complete the payment form
- Pay participants
